# Evaluation of wetting ability of five new saliva substitutes on heat-polymerized acrylic resin for retention of complete dentures in dry mouth patients: a comparative study

**DOI:** 10.11604/pamj.2017.27.185.9098

**Published:** 2017-07-10

**Authors:** Abdul Habeeb Bin Mohsin, Varalakshmi Reddy, Praveen Kumar, Jeevan Raj, Siva Santosh Babu

**Affiliations:** 1 Department of Prosthodontics, Sri Sai College of Dental Surgery, Vikarabad, India; 2 Department of Prosthodontics, MNR Dental College and Hospital, Sangareddy, India

**Keywords:** Wettability, xerostomia, retention, denture, saliva

## Abstract

**Introduction:**

The aim of this study was to evaluate & compare the wetting ability of five saliva substitutes & distilled water on heat-polymerized acrylic resin. Contact angle of the saliva substitute on denture base can be taken as an indicator of wettability. Good wetting of heat-polymerized acrylic resin is critical for optimum retention of complete dentures.

**Methods:**

Two hundred & forty samples of heat-polymerized acrylic resin were fabricated using conventional method. 240 samples divided into 6 groups with 40 samples in each group. Advancing & Receding contact angles were measured using Contact Angle Goniometer & DSA4 software analysis.

**Results:**

Anova test was carried out to test the significance in difference of contact angle values in the six groups. The mean of advancing angle values & mean of receding angle values of all the six groups has shown statistically significant difference between the groups. The mean of angle of hysteresis values of all the six groups are statistically not significant between the groups. A multiple comparison using Bonferroni’s test was carried out to verify the significance of difference between the contact angles in a pair of groups. Statistically significant difference was seen when Aqwet (Group II) was compared to Distilled water (Group I), Wet Mouth (Group III), E-Saliva (Group IV), Biotene (Group V), and Moi-Stir (Group VI).

**Conclusion:**

The contact angles of five saliva substitutes and distilled water were measured and compared. Group II (AQWET) has the lowest advancing and receding contact angle values and the highest angle of hysteresis on heat-polymerized acrylic resin. Based on contact angle values, Group II (AQWET) has the best wetting ability on heat-cured acrylic resins. The ability of saliva to wet the denture surface is one of the most important properties for complete denture retention in dry mouth cases.

## Introduction

Retention of complete dentures can be affected by physical, physiological, psychological, mechanical, and surgical factors. The physical factors are further divided into adhesion, cohesion, interfacial surface tension, and atmospheric pressure. These physical factors operate in the fluid film between the denture base and the mucosa. The salivary mucins possess rheological properties that include elasticity and adhesiveness, which aid in retention of dentures. Saliva is essential to the function and protection of the oral cavity and contiguous gastrointestinal epithelium. Common functions of the fluid component of the salivary secretions include cleansing and lubrication of oral soft and hard tissues, solubilization and bolus formation of food, facilitation of taste perception, mastication and speech and retention of removable prosthesis [1]. Saliva has many mechanical and chemical functions and is fairly, or sensitive parameter of certain bodily functions. The patients who wear complete denture prosthesis depend on this oral fluid to provide retention and at the same time to prevent friction between the dentures and the mucosa. Xerostomia is the subjective sensation of dryness of oral mucous membrane with the objective evidence of significantly decreased salivary flow. Xerostomia could be the result of radiation treatment for oral cancer or due to the presence of systemic conditions like rheumatoid conditions, sjogren’s syndrome, diabetes mellitus, parkinson’s disease and dysfunctions of the immune system like HIV/AIDS. Xerostomic patients have difficulty with chewing, swallowing, and speech. Dryness of the oral mucosa renders it more susceptible to irritation and epithelial atrophy, leading to possible inflammation, fissuring, and ulceration. The wearing of a dental prosthesis may cause discomfort. When the discomfort of oral mucosa is combined with tasting problems, xerostomic patients lose weight. Qualitative and quantitative deficiency of the saliva causes an unhealthy and painful oral environment [2]. Denture wearing may become difficult because dry mouth can significantly add to the problem of retaining and eating with the dentures, which invariably become loose.

Kawazoe and Hamada (1978) [3] stated that maxillary denture retention was influenced by the salivary volume between the denture base and the mucous membrane. An optimum intervening salivary volume at which the greatest retention was developed, was also observed. It was observed from determination of electric resistance of palatal mucous membrane that inward and outward flow of intervening saliva was greater in the denture with poor retention than the one with good retention. Moreover when the salivary volume between the denture base and mucous membrane was optimum, the salivary flow under the denture was minimum [4–6]. The liquid-joint model is proposed by most authors. According to Stanitz (1948) [7] the retention force is a function of saliva surface tension, liquid film thickness, surface of contact, and liquid-denture contact angle. Others have analyzed the role of the viscosity of this liquid. Bla’hova’ and Neuman (1971) [8] said viscosity of saliva helps prevent the dislodgment of denture. Viscosity becomes an important factor of retention in the initial phase [9, 10]. Replacement of saliva by a fluid other than saliva has been proposed as a possible treatment in relieving subjective complaints of xerostomia for more than three decades. Water can be used as a saliva replacement, but it is known that water does not moisten and lubricate the oral mucosa and teeth adequately. Therefore saliva substitutes containing thickening agents for longer relief and increased moistening and lubrication of the oral surfaces have been developed. These are agents formulated as solutions, sprays or gels and have multiple contents including carboxymethylcellulose, electrolytes and flavoring agents. Ideally, saliva substitutes should be pleasant in taste and odor, non-toxic, non-addictive, economical and must exhibit good wetting of the tissue surface of the denture. Good wetting of the heat-polymerized acrylic resin by the saliva substitute is critical for optimum retention of the complete dentures. For good adhesion of the denture to the supporting tissues, the saliva or saliva substitute must flow easily over the entire surface to ensure wetting of the adherent surface. The contact angle of the saliva substitute on the denture can be taken as an indicator of the wettability – the smaller the contact angle, the greater the wettability or the contact angle is a useful inverse measure of wettability. Taking into the consideration importance of wetting of acrylic denture base by saliva substitutes in xerostomia patients, this study was undertaken to evaluate and compare the wettability of five available saliva substitutes and distilled water on heat-polymerized acrylic resin.

## Methods

**Figure 1:**
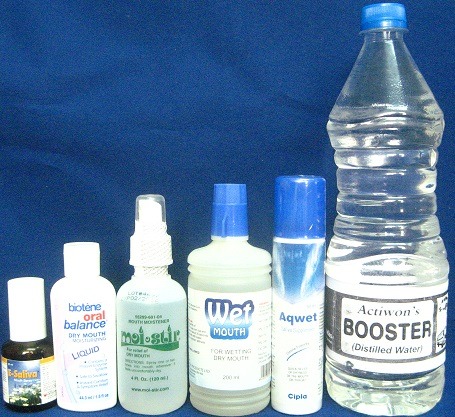
Five salivary substitutes & distilled water

Five saliva substitutes and Distilled water were used in this study (Figure 1). They are 1. Distilled water (Actiwon’s), 2. Aqwet - “Saliva supplement”, (Cipla Limited), 3. Wet Mouth - “For wetting dry mouth”, (ICPA Health Products Ltd.), 4. E-Saliva - ‘Mouth Spray’, (Entod Pharma Ltd.), 5. Biotene - “Oral balance”, (GlaxoSmithKline, USA), and 6. Moi-Stir - ‘Mouth moistener´, (Pharmascience Inc.). Two hundred & forty acrylic resin samples were made using DPI (Dental Products of India) Heat cure acrylic resin.

**Figure 2:**
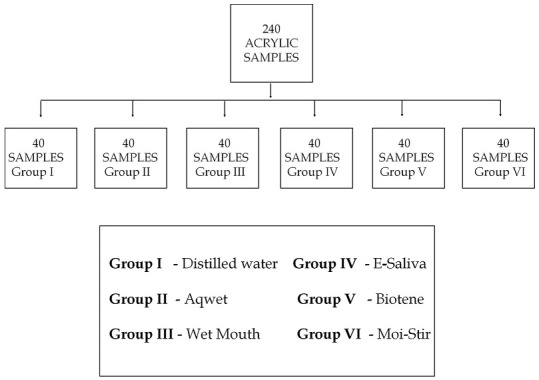
Sample size and distribution

**Sample distribution**: Two hundred & forty samples of heat-cured acrylic resin were made. 240 samples were divided into 6 groups each containing 40 samples (Figure 2).

**Figure 3:**
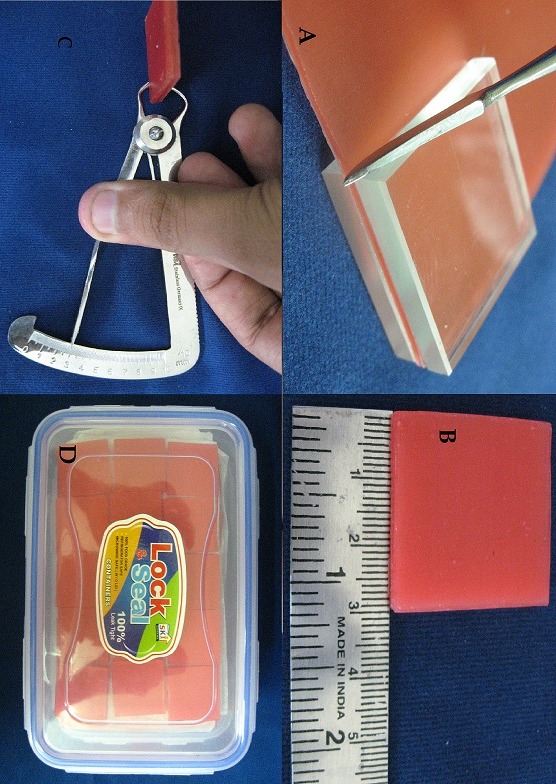
Sample preparation: (A) cutting of wax sample using carver; (B) wax sample measuring 30x30mm; (C) measuring thickness of wax sample using wax gauge; (D) samples stored in air-tight container

**Preparation of samples**: Two hundred and forty samples of heat-cured acrylic resin were made by the following method:

**Wax samples fabrication**: Modelling wax of 1.5 mm thickness was taken to get total thickness of 3 mm by placing one sheet over the other. Total thickness of 3 mm was taken to compensate for the loss of acrylic during the finishing procedure and to get uniform thickness of 2 mm in the final acrylic samples. Two square glass plates measuring 30 x 30 mm were used to cut the wax strips along all the sides using a sharp carver. The wax samples were checked for uniformity using wax gauge. The storage of samples was done in plastic air-tight containers (Figure 3).

**Figure 4:**
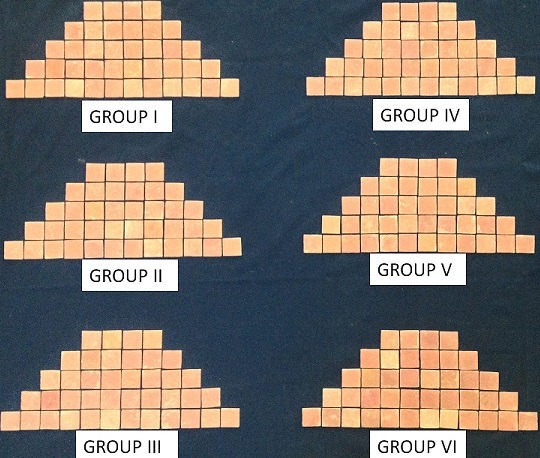
240 heat-cure samples divided into 6 groups

**Heat-cured acrylic resin samples fabricated**: Dental plaster was used to invest the wax samples in varsity denture flasks. A heat-activated conventional acrylic denture base resin material (Dental Products of India) was used. Processing was done according to the manufacturer’s instructions. Two hundred & forty samples were made by following the above method (Figure 4). The samples were stored in normal water for one day. Samples finishing was done to get an even thickness of 2 mm using flat cherry stones and sandpaper as to simulate clinical conditions. Surface to be tested (tissue surface) was not finished to simulate clinical practice. The samples were finished on the other side (polished surface) manually using sandpaper, to get a flat surface. First the cleaning of samples was done for 5 mins with soft cotton and household soap immersed in water and then the samples were rinsed well in running water. To remove any soap residues they were cleaned with spirit. Then they were immersed in ultrasonic cleaner for 15 mins. With the help of soft tissues the samples were dried. To verify the effectiveness of finishing the samples were then viewed under scanning electron microscope at 2000× magnification.

**Preparing the samples**: The samples were placed in oven. Then, the samples were oven dried at 44°C for 30 min and then cooled to a temperature of 22°C by room air conditioner.

**Figure 5:**
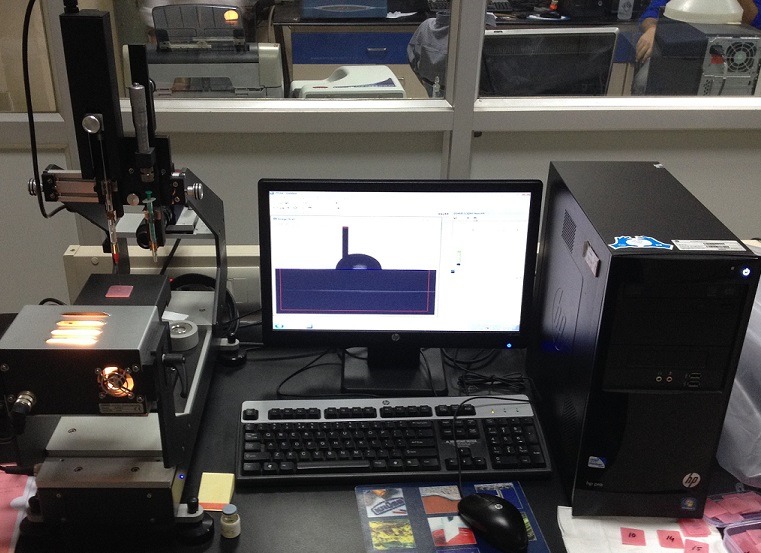
Advancing and receding contact angles were measured using contact angle goniometer DSA25 and software DSA4

**Figure 6:**
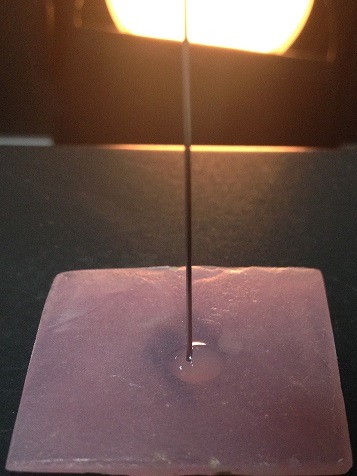
Measurement of advancing and receding contact angles on the sample using goniometer syringe

**Figure 7:**
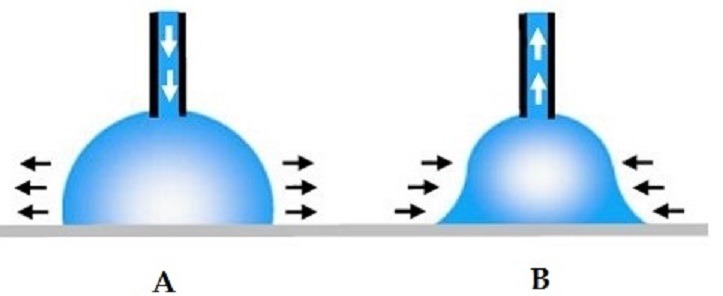
Illustration of advancing and receding contact angles: (A) advancing angle; (B) receding angle

**Contact angles measurement**: The advancing and receding contact angles were measured using contact angle goniometer and software DSA4 (Figure 5). All the 240 samples were divided into six groups with 40 samples in each group. Distilled water was used in Group I; AQWET in Group II; WET MOUTH in Group III; E-SALIVA in Group IV; BIOTENE in Group V; and MOI-STIR in Group VI. A pre-cleaned, oven-dried glass syringe was filled with distilled water. It was graduated in microlitres; therefore, the liquid used for each drop could be standardized. The acrylic sample was held with tweezers only on the sides, taking care not to touch the tissue surface of the sample. The sample was placed at the centre of the table just below the needle of the syringe. The software program DSA4 was used to measure the advancing and receding contact angles. After measuring the advancing contact angles, the drop was drawn backward through the needle and then receding contact angles were measured (Figure 6). After the values were obtained, the sample was removed and a new sample was placed. The procedure was repeated for 40 samples in the first group. Later, the above procedure was repeated for all the samples in the six groups, and measurements were recorded (Figure 7). Methods adopted for statistical analysis are Anova test and Bonferroni’s test. Anova was carried out to test the significance in difference of contact angle values in the six groups. Bonferroni’s multiple comparison test was carried out to verify the significance of difference between the contact angles in a pair of groups.

## Results

This study was done to compare wettability of distilled water and five saliva substitutes on heat cure acrylic resin using contact angle goniometer. Measurement of advancing and receding contact angles of distilled water and five saliva substitutes (namely Aqwet, Wet Mouth, E-Saliva, Biotene & Moi-Stir) was carried out for 240 acrylic samples. The study comprised of 240 acrylic samples of which 40 samples were used to measure advancing and receding contact angles of Distilled water which were considered as Group I, 40 samples were used to measure advancing and receding contact angles of Aqwet which were considered as Group II, 40 samples were used to measure advancing and receding contact angles of Wet Mouth which were considered as Group III, 40 samples were used to measure advancing and receding contact angles of E-Saliva which were considered as Group IV, 40 samples were used to measure advancing and receding contact angles of Biotene which were considered as Group V, 40 samples were used to measure advancing and receding contact angles of Moi-Stir which were considered as Group VI. The angle of hysteresis was calculated as the difference between the advancing and receding contact angle values. The data was subjected to analysis using IBM SPSS 19.0 PACKAGE. A p-value of (< 0.05) was considered statistically significant. Means of contact angle values were as follows (Table 1). Anova test was carried out to test the significance in difference of contact angle values in the six groups. Anova was carried out to test the significance in difference of contact angle values in the six groups: The mean advancing angle of distilled water (group I) was 89.1350, aqwet (group II) was 76.6750, wet mouth (group III) was 88.9050, E-saliva (group IV) was 83.6200, biotene (group V) was 87.5850, moi-stir (group VI) was 86.0550. There was a significant difference in the mean of advancing values among the six groups (p-value < 0.05). The mean receding angle of distilled water (group I) was 82.4050, aqwet (group II) was 70.6225, wet mouth (group III) was 83.6125, E-saliva (group IV) was 79.3725, biotene (group V) was 82.0075, moi-stir (group VI) was 81.0750. There was a significant difference in the mean of receding angle values among the six groups (p-value < 0.05). The mean angle of hysteresis of distilled water (group I) was 6.7300, aqwet (group II) was 5.9875, wet mouth (group III) was 5.2925, E-saliva (group IV) was 4.2475, biotene (group V) was 5.5775, moi-stir (group VI) was 4.9800. The mean of angle of hysteresis values are not significant between the mean values of six groups (p-value > 0.05). Anova analysis shows that mean of advancing angle values & mean of receding angle values of all the six groups has shown statistically significant difference between the groups. The mean of angle of hysteresis values of all the six groups are statistically not significant between the groups (Table 2). Multiple comparison Bonferroni’s test was applied out to verify the significance of difference between the contact angles in a pair of groups there is statistically significant difference was seen when Aqwet (Group II) was compared to Distilled water (Group I), Wet Mouth (Group III), E-Saliva (Group IV), Biotene (Group V), and Moi-Stir (Group VI).

**Table 1:** Contact angle measurement mean values

	Groups	N	Mean	SD	Minimum	Maximum
**Advancing Angle**	Distilled water	40	89.1350	11.39947	64.00	110.50
	Aqwet	40	76.6750	7.51121	63.50	101.20
	Wet Mouth	40	88.9050	10.32470	66.20	110.00
	E-Saliva	40	83.6200	7.97079	69.60	103.60
	Biotene	40	87.5850	7.53503	73.70	105.50
	Moi-Stir	40	86.0550	9.46616	66.30	104.80
**Receding Angle**	Distilled water	40	82.4050	11.74337	58.00	103.00
	Aqwet	40	70.6225	8.15354	28.70	82.50
	Wet Mouth	40	83.6125	11.13240	62.20	104.80
	E-Saliva	40	79.3725	7.25534	62.90	93.40
	Biotene	40	82.0075	6.84347	68.90	97.30
	Moi-Stir	40	81.0750	10.47375	56.50	101.40
**Angle of Hysteresis**	Distilled water	40	6.7300	6.46474	0.10	33.50
	Aqwet	40	5.9875	7.21122	0.30	34.80
	Wet Mouth	40	5.2925	4.62925	-3.80	16.70
	E-Saliva	40	4.2475	2.96622	1.00	14.80
	Biotene	40	5.5775	4.43381	-8.10	15.70
	Moi-Stir	40	4.9800	4.31777	-12.30	15.10

**Table 2:** Anova test (p value < 0.05)

		Sum of Squares	df	Mean Square	F	Sig,
**Advancing Angle**	Between Groups	4428.097	5	885.619	10.566	0.000
	Within Groups	19612.479	234	83.814		
	Total	24040.576	239			
**Receding Angle**	Between Groups	4488.585	5	897.717	10.021	0.000
	Within Groups	20962.095	234	89.582		
	Total	25450.680	239			
**Angle of Hysteresis**	Between Groups	145.323	5	29.065	1.074	0.375
	Within Groups	6330.669	234	27.054		
	Total	6475.992	239			

## Discussion

The wearing of dental prosthesis causes discomfort in xerostomic patients. A lack of salivary buffering action also leads to increased risk of caries. Qualitative and quantitative deficiency of the saliva causes an unhealthy and painful environment. In addition, saliva plays an important role in the retention of complete dentures and protecting oral health. A factor that would affect the magnitude of contact angle of a fluid on a solid surface is the roughness of the adherent surface, which differs with respect to the solid. Surface roughness, even in test specimens of the same group of denture base material, was an uncontrollable variable. The variability in surface roughness of denture base materials must be considered when contact angles are evaluated. Ozden et al (1999) [11] said that glow discharge plasma altered the surfaces of the acrylic resin and increased the wettability as shown both by X-ray photoelectron spectroscopic and contact-angle measurements, and plasma treatment seemed to offer a durable (at least up to 60 days) wettability. Gesser and Castaldi (1971) [12] said that the vacuum discharge method appeared to give a high percentage of improvement in retention but, since the durability of the surface treatments was not tested, no real comparison can be made.

Contamination of the studied surfaces may produce a change in the water surface tension that, in turn, would induce an error in the measured contact angle values. The origin of this contamination may be of chemical nature (migration of the residual monomer from the polymer bulk to the surface) or of microbial nature (formation of metabolites). However, in the application of angle measuring techniques, the liquid drop was left in contact with the polymer surface for less than 2 min. Moreover, the extremely careful rinsing procedure made it highly improbable that either chemical or microbial contamination occurred. Surface heterogeneity may play its role in increasing contact angle hysteresis. In this case, the advancing contact angle would depend on the fraction of the surface occupied by a low surface-energy phase; the receding angle would be influenced by a high surface-energy phase. O’brien & Ryge (1965) [13] said that the wettability was excellent in comparison with an untreated denture. The best wettability was found on the tissue side of the dentures which is the critical area for retention. Murray (1988) [14] investigated the effect of surface treatment on poly methyl methacrylate when exposed in the mouth. They indicated that it is difficult, if not impossible, to hydroxylate the surface of PMMA by an aqueous treatment. The sole effect of low-pressure discharge seems to be one of decontamination. Artificial saliva products are useful agents for the palliative treatment of xerostomia at present. Saliva substitutes are divided into two groups: carboxymethylcellulose and mucin-based saliva substitutes. Mucin based saliva substitutes have been proved to show better wettability than carboxymethylcellulose-based saliva substitutes, but they are derived from porcine derivatives, mainly the gastric mucin, and therefore are likely to be objectionable to the Indian population. Therefore, saliva substitutes used in the study contained carboxymethylcellulose, which imparts lubrication and viscosity [2, 15, 16]. A. Vissink et al (1986) [15] said that the contact angles of water and carboxymethylcellulose or mucin containing saliva substitutes were significantly lower than those of human whole saliva on ground, polished enamel. The contact angle of water on human mucosa was significantly higher than that of human whole saliva. The contact angles of the carboxymethylcellulose-preparations and human whole saliva were comparable on human mucosa. In study, done by Aydin et al (1997) [2] both types of artificial salivas (mucin-based and carboxymethylcellulose-based substitutes) had better wetting properties on denture base resin than the natural saliva. The human saliva had the highest contact angle. Oh et al (2008) [17] said that carboxymethylcellulose-based artificial saliva has significantly better effects in patients with very severe dry mouth whose functional salivary gland capacity was severely compromised. Carboxymethylcellulose-based artificial saliva decreased the patients’ discomfort during the night as well as during the day. Their quality of life may also increase following the use of carboxymethylcellulose-based artificial saliva. To produce adequate adhesion of a denture to the supporting tissues, saliva must flow easily over the entire surface to ensure wetting of the adherend surface. The fundamental requirement for good adhesive performance is intimate molecular contact between the adhesive and the adherend. The extent to which the adhesive will wet a surface depends on the viscosity of the adhesive, the shape of the irregularities on the surface of the adherend, and on the contact angle at which the adhesive meets the adherend surface. Since the tendency for the liquid to spread increases as angle decreases, the contact angle is a useful inverse measure of spreadability or wettability. The contact angle observed when a liquid boundary advances for the first time over a dry smooth surface is called the advancing contact angle, while the contact angle observed when a liquid boundary recedes from a previously wetted surface is called the receding contact angle. Contact angles are characteristic constants of liquid/solid systems. When contact angles are determined, the liquid and solid surfaces must be uncontaminated by extraneous impurities, and the liquid and solid must be mutually insoluble and not react chemically. Cleaning methods that are extremely effective in removing foreign contaminants are needed for contact angle measurements.

The existence of continuous salivary layer is extremely important for two reasons: 1) Salivary contact angle on the mucosa i.e. equal to zero. 2) The volume of the saliva beneath the denture may not be considered as constant because of the salivary flow. The contact angle hysteresis and denture geometry at the meniscus contact lines are determinant factors of denture retention [9, 10]. The significant differences in the contact angle values must be analyzed in terms of the advancing, receding angles and contact angle hysteresis induced by these surfaces. Contact angle hysteresis is influenced by surface heterogeneity, surface roughness, surface deformation and chemical contamination of water. In addition, contact angle hysteresis of polymer surfaces can be induced by the mobility and reorientation of surface polymeric chains. The presence of liquid in contact with a solid may provoke the reorientation of polymer surface groups, leading to contact angle hysteresis. Equilibrium contact angle has been regarded as related to denture comfort, and denture retention is more related to contact angle hysteresis. Theoretical considerations and experimental results clearly demonstrate that, with the exception of some specific cases such as perfectly wettable solids, the contact angle of the advancing liquid front on a dry solid surface (advancing contact angle) is different than the receding contact angle [9]. When the liquid front recedes on a solid surface, the dewetting mechanism produces at first a contact angle variation and then a displacement of the liquid solid contact line. When, instead of pure liquids, solutions containing different surface-active agents (such as surfactant or proteins) are used in contact angle measurements, adsorption of these molecules at the liquid-solid interface induces an important hysteresis. Force required for dislodging the denture vertically denoted as F max

$$Fmax=mg\frac{\text{Cos }\partial \text{R}}{\text{Cos }\partial \text{A}}$$

Where *mg* is the weight of the denture, ∂R is the receding angle and ∂A is the advancing angle. From this equation, it is clear that force required for separating the two surfaces increases with increase in hysteresis angle. The capillary force, which helps restrain any dislodging force on the denture, is increased by complete wetting of the surface, high surface tension of the saliva and large tissue contact area of the denture. The capillary force F, responsible for retention of a denture, can be expressed by the following equation:

$$F=\frac{\gamma^{∼}\text{A(cos }\partial \text{1 + cos }\partial \text{2)}}{\text{Dg}}$$

Where γ˜ is the surface tension of saliva, A is the area of tissue surface of the denture, ∂1 is the advancing contact angle, ∂2 is the receding contact angle, *D* is the film thickness, and *g* is the gravitational force. Thus, contact angle hysteresis and denture geometry at the meniscus contact line are the determinant factors of denture retention. A large number of important conditions influencing denture retention in mouth may be taken into account while choosing denture base materials and denture shape. For example, retention might be improved in cases of denture base surfaces with high values of advancing angles and low values of receding angles. Good wetting of the acrylic denture base resin by saliva substitutes is of clinical importance. The quality of life for xerostomia patients may be improved by the use of a suitable saliva substitute. According to wettability studies the measurement of contact angles, the primary data indicates the degree of wetting when a solid and liquid come into contact. Small contact angles (< 90°) correspond to high wettability, while large contact angles (> 90°) correspond to low wettability. More specifically, a contact angle less than 90° indicates that wetting of the surface is favorable, and the fluid will spread over a large area on the surface, it is considered as hydrophilic; while contact angles greater than 90° generally means that wetting of the surface is unfavorable so the fluid will minimize its contact with the surface and form a compact liquid droplet, it is considered as hydrophobic. For example, complete wetting occurs when the contact angle is 0°, as the droplet turns into a flat puddle [18]. Advancing contact angle is defined as the angle that a liquid drop forms on a dry solid surface. Receding angle is formed when the liquid recedes on the previously wet solid surface. The difference between the advancing angle and the receding angle is called hysteresis. Angle of Hysteresis is indicative of potential for greater wetting as the value increases. As the difference of advancing and receding angle increases angle of hysteresis increases which is proportional to both the values. The difference window of advancing and receding angle values determines angle of hysteresis so that the potential for wetting of the heat cure acrylic resin can be evaluated based on it. Contact angle hysteresis is influenced by surface heterogeneity, surface roughness, surface deformation and chemical contamination of water.

This study was done to compare wettability of distilled water and five saliva substitutes on heat cure acrylic resin using contact angle goniometer. The mean advancing angles of all the groups were compared. Aqwet showed lowest contact angle which indicates the wettability is more on heat polymerized acrylic resin. Good wetting of the heat polymerized acrylic resin by the saliva substitute is critical for optimum retention of the upper complete denture. Statistically significant difference was seen when the mean advancing angle measurement of aqwet (group II) (76.6750) was compared to the remaining five groups in a pair wise test. This could be because the contact angle of aqwet is less than remaining five groups as lesser the contact angle more the wettability. Comparison result of aqwet with distilled water and wet mouth are similar to those found by Sharma and Chitre [19]. Mathrawala N.R et al (2011) [20] in their study found that wettability of Aqwet was better than other substitutes used in their study. The mean receding angles of all the groups were compared for receding angle. Aqwet showed lowest contact angle which indicates the wettability is more on heat polymerized acrylic resin. Statistically significant difference was seen when the mean receding angle measurement of aqwet (group II) (70.6225) was compared to the remaining five groups in a pair wise test. This could be because the contact angle of aqwet is less than remaining five groups as lesser the contact angle more the wettability. Comparison result of aqwet with distilled water and wet mouth are in correlation with those found by Sharma and Chitre [19]. Bikash et al (2010) [21] suggested use of aqwet and wet mouth saliva substitutes containing thickening agents for longer relief and increased moistening and lubrication of the oral surfaces in xerostomia patients. The mean angle of hysteresis of all the groups was compared for angle of hysteresis. Aqwet showed highest angle of hysteresis among saliva substitutes which indicates the wettability is more on heat polymerized acrylic resin. The mean values of all six groups were compared with each other in a pair of groups and the values for mean difference were obtained for each comparison. Statistically no significant difference was seen.

## Conclusion

Saliva plays a substantial role in the retention of complete dentures. To create sufficient denture adhesion to the bearing tissues, saliva must wet the contact surfaces and flow easily over the surfaces. The ability of saliva to wet the denture surface is one of the most important properties for complete denture retention. The contact angle of a liquid on a substrate is an indication of its wettability: the smaller the angle, the greater the wettability. With a contact angle of 0°(degree) complete wetting is said to occur; therefore the retention would be expected to be greatest with an angle of 0°(degree). The purpose of this in vitro study was to determine the wetting ability of different artificial saliva substitutes on heat-polymerized acrylic resin samples using contact angle measurements, to present suitable saliva substitute for best wetting in dry mouth (xerostomic) denture wearers. The contact angles of distilled water and five saliva substitutes were measured and compared. Despite several limitations of this study, the following conclusions can be drawn: Group II (AQWET) has the lowest advancing and receding contact angle values and the highest angle of hysteresis on heat-cured acrylic resin (DPI heat-cured denture material). Based on contact angle values, Group II (AQWET) has the best wetting ability on heat-cured resin acrylic dentures fabricated with DPI heat-cured denture material.

### What is known about this topic

- Flow and contact angles plays important role in wetting ability of saliva substitutes;
- Necessary need of saliva substitute for the dry mouth denture patients;
- There is diminished production of natural saliva in xerostomia patients (various medical reasons) and cancer patients.

### What this study adds

- This study presented suitable saliva substitute for best wetting in dry mouth (xerostomic) denture wearers;
- In depth study of flow and wetting of saliva to the oral bearing tissues;
- This study adds to the use of best available saliva substitute for the cancer patients.

## Competing interests

The authors declare no competing intererst.
